# Fatal Isolated Right Ventricular Rupture Without External Chest Injury in a Young Driver: Forensic Autopsy Findings After a One-Sided Vehicle Collision

**DOI:** 10.34172/aim.34401

**Published:** 2025-09-01

**Authors:** Yusuf Atan, Mehmet Doğan, Ferah Karayel, İbrahim Üzün

**Affiliations:** ^1^Council of Forensic Medicine, Ministry of Justice, Republic of Turkiye, Istanbul, Turkiye; ^2^Forensic Medicine Department, Bilecik Seyh Edebali University Faculty of Medicine, Bilecik, Turkiye; ^3^Forensic Medicine Department, Istanbul University Cerrahpasa Faculty of Medicine, Istanbul, Turkiye

**Keywords:** Blunt cardiac injury, Forensic medicine, Trauma, Traumatic death, Vehicle collision

## Abstract

Traumatic deaths are common, with cardiac trauma affecting 7‒12% of patients with thoracic injuries. Blunt cardiac injury (BCI), although rare, is associated with a high mortality rate. This report presents a case of blunt cardiac rupture (BCR) observed at autopsy despite the absence of external chest trauma, suggesting the presence of severe internal injuries. A 19-year-old male was found dead in his vehicle which had collided with a wall. At the crime scene investigation, external examination revealed no substantial chest wall injuries in the individual despite significant damage to the vehicle. Autopsy revealed a 2-cm rupture of the right ventricle (heart), accompanied by 400 cc of partially coagulated blood in the pericardial cavity, consistent with cardiac tamponade. Pregabalin was detected in the toxicology analysis, but not in lethal concentrations. Traffic accidents are a major cause of BCI, typically resulting from compression of the heart between the thoracic structures during high-energy impacts. BCR is particularly fatal and often results in rapid death before arrival to the hospital. The absence of external trauma in the current case underscores the need for thorough internal examination in trauma-related deaths.

## Introduction

 Traffic accidents are the leading cause of trauma-related deaths in individuals aged between 5 to 44 years.^1,2^ With a global increase in car traffic, the frequency of such accidents has risen.^3^ Every year, around 1.35 million people die in road traffic accidents worldwide.^4^ Cardiac trauma is observed in 7%‒12% of patients with thoracic trauma. In addition to injuries affecting the whole body, blunt or penetrating chest injuries are frequently observed in drivers or passengers involved in vehicle collisions. Although penetrating chest trauma can have a variety of causes, the most common cause of blunt chest trauma is a motor vehicle collision.^1^ Cardiac traumas can result from two main mechanisms: penetrating and blunt injuries. Blunt injuries may occur due to various reasons, such as being kicked by animals, falls, physical assaults, and traffic accidents.^5-7^ Cardiac injuries, especially rupture, due to a blunt traumatic injury are very rare but can have a high mortality rate. The incidence of trauma-related hospital admissions is approximately 0.16‒2%; this rate is likely to be higher when cases of individuals who die before reaching the hospital are considered.^8-14^

 Blunt cardiac injury (BCI) most commonly occurs due to a direct blow to the chest, often resulting from sudden acceleration towards the steering wheel in road traffic accidents. The heart, located between the sternum and thoracic vertebrae, is compressed within the thorax due to this sudden impact. High-energy blunt traumas can cause injuries to the valves, the conduction system or myocardial regions and can range from asymptomatic myocardial contusions to lethal cardiac rupture.^15^ Such traumas have a high mortality rate due to associated hemorrhages and/or complications such as arrhythmia. Although BCI is a common complication of chest trauma in traffic accidents, blunt cardiac rupture (BCR) resulting from accident related trauma is comparatively rare.^16,17^ The current case study shows that cardiac rupture may occur as a result of high-impact trauma in young individuals without any external traumatic lesions or chest wall injuries. We aimed to share this finding with professionals working in the fields of emergency medicine, cardiology and forensic medicine.

## Case Report

**Figure 1:**
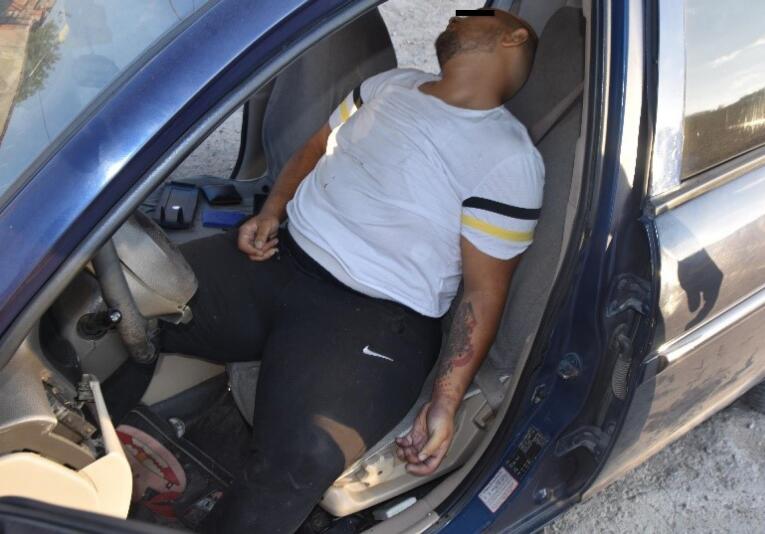
Image from the Crime Scene Investigation (showing the deceased driver in the seat and the broken steering wheel)

 A 19-year-old male with an unknown medical history was found dead in the driver’s seat of a vehicle (Figure 1). The vehicle had struck the curb on the side of the road and travelled some distance over the curb, followed the gravel road on the right side, and eventually came to rest after crashing into a utility pole and a wall of an animal farm.

###  Crime Scene Investigation Findings

 Paint marks, dents, and breakage marks were observed on the wall and the concrete electric pole due to the impact from the car. Inside the vehicle, a male individual was seen lying on his back, seated with his head leaning on his right shoulder, wearing a white t-shirt over black sweatpants, with his right leg resting on the gear shift. Examination of the vehicle’s interior revealed that the bottom part of the steering wheel was broken and bent inwards, the right airbag had deployed; moreover, the seat belt was buckled behind the individual and had no protective feature. The vehicle’s gauges were at the “0” position, the car was in the fifth gear, and the handbrake was down.

###  Examination of the Deceased

 The body was transported to the hospital morgue for further forensic investigation. Toxicological analysis of the blood collected from the body revealed 4.4 mg/dL ethanol and 950 ng/mL pregabalin. External examination at the hospital morgue did not reveal any traumatic findings that could explain the cause of death, prompting the decision to perform an autopsy.

###  Autopsy Findings

**Figure 2:**
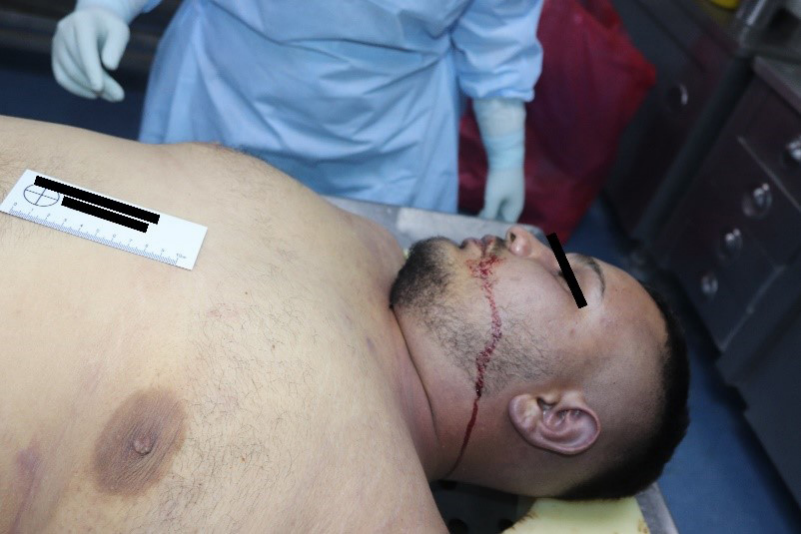
Autopsy Image-1 (external view of the chest wall)

**Figure 3:**
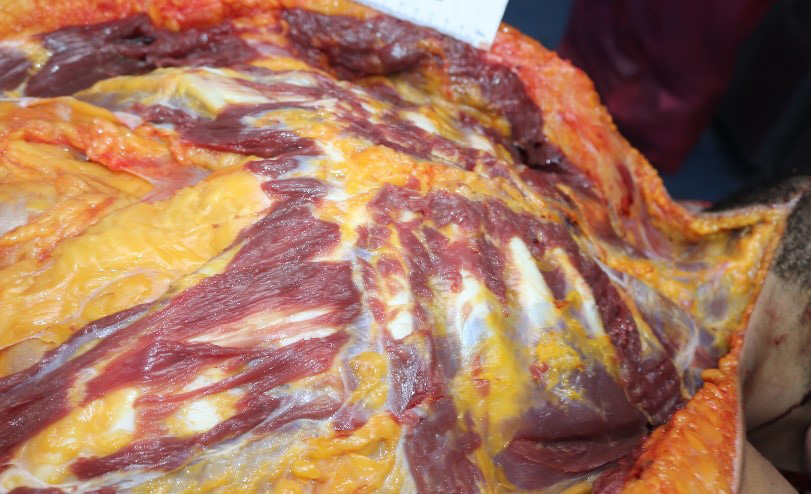
Autopsy Image-2 (internal view of the chest wall)

**Figure 4:**
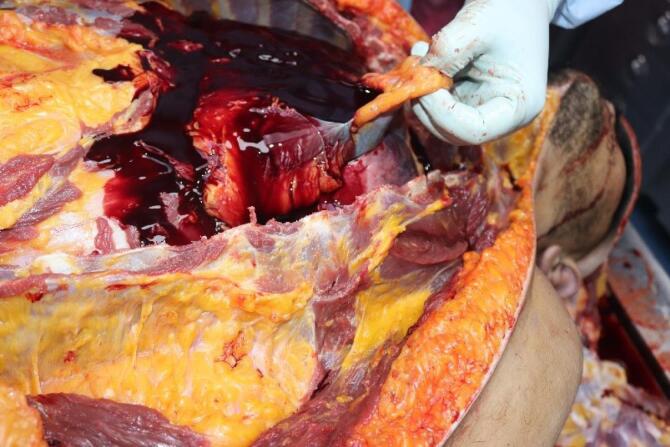
Autopsy Image-3 (cardiac tamponade)

**Figure 5:**
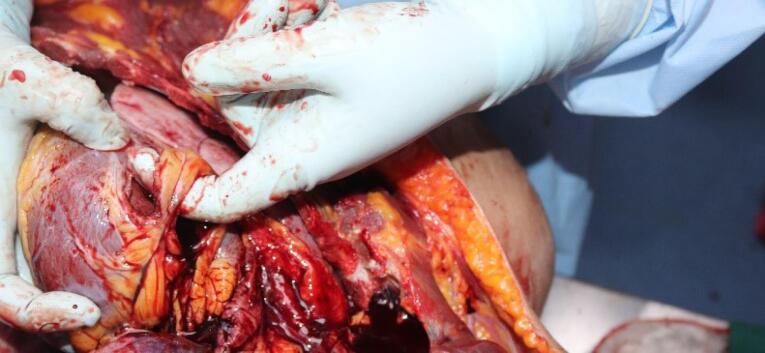
Autopsy Image-4 (cardiac rupture-anatomic view)

**Figure 6:**
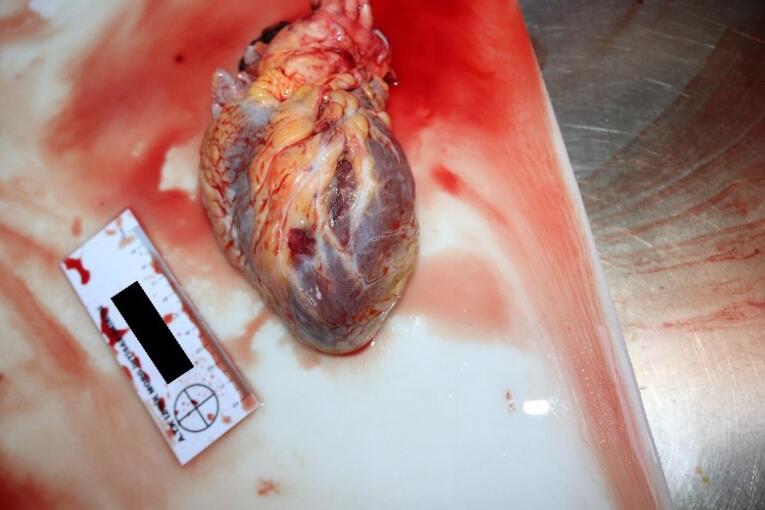
Autopsy Image-5 (cardiac rupture-opposite view)

 The medicolegal autopsy conducted at the Council of Forensic Medicine, Izmir Group, Morgue Specialization Department, revealed that the deceased was 178 cm tall and weighed 115 kilograms. The external examination identified minor abrasions at the lower extremities. No traumatic findings were noted in the chest (Figure 2) or other areas of the body. Internal examination showed no ecchymosis or hematoma in the soft tissues or the muscles of the chest wall (Figure 3). Additionally, no bruising was detected in the pericardium, which remained intact. Upon opening the pericardial cavity, approximately 400 cc of partially coagulated blood (Figure 4) and a 2-cm rupture in the right ventricle of the heart were observed. The rupture was associated with the ventricular cavity. A 0.6 × 0.6 cm contusion area just below and 0.2 cm lateral to this rupture was also found (Figures 5 and 6). The coronary artery lumens were open, ventricular wall thickness and heart valve dimensions were normal. However, hemorrhage and tissue destruction were noted in the rupture area within myocardial sections. No significant findings were found in histopathological examinations. The skeletal system was intact including the sternum and ribs. No alcohol (ethanol or methanol) was detected in the toxicological analysis of the blood and intraocular fluid samples taken at the autopsy. A concentration of 660 ng/mL of pregabalin was found in the blood. The cause of death was determined to be hemorrhage and cardiac tamponade resulting from cardiac rupture due to blunt chest trauma.

## Discussion

 Blunt chest traumas may result from four key mechanisms of injury: direct impact, thoracic compression, rapid acceleration/deceleration, and blast-related trauma. Among these, injuries caused by direct impact tend to be less severe, typically affecting the soft tissues of the chest wall, leading to hematomas or abrasions. In some instances, localized trauma may involve the bony structures of the thorax, such as the rib or sternal fractures and sternoclavicular dislocations. In rare cases, the force of impact can be transmitted through the chest wall, leading to significant damage to the internal organs, including the heart, lungs, or major mediastinal vessels. Thoracic compression injuries occur when intrathoracic structures are forcibly compressed against a fixed anatomical structure, such as the chest wall or spine, resulting in contusions or ruptures of internal organs. This mechanism can lead to pulmonary contusion, laceration of lung parenchyma, pneumothorax, hemothorax, tracheobronchial fractures, or even diaphragmatic rupture. Acceleration/deceleration injuries are of particular concern due to the shearing forces they generate, which cause direct compression against fixed anatomical points. These injuries are among the most common and have a high mortality, often resulting in severe tracheobronchial disruptions, myocardial contusions, and catastrophic damage to the aorta and diaphragm.^18^ The blast-related trauma mechanism entails the transfer of high energy to the abdomen and lower extremities, which can lead to cardiac rupture through a water hammer effect. The rapid increase in hydrostatic venous pressure is transmitted to the right atrium, potentially causing injury. An additional important factor for cardiac rupture following blunt trauma is the timing of the injury in relation to the cardiopulmonary cycle, which can also affect the type of injury. The cardiac chambers are highly susceptible to rupture when they are fully expanded, with end diastole being the phase of highest risk for ventricular rupture. Cardiac rupture resulting from compression or direct osseous penetration is often associated with significant thoracic or extrathoracic injuries; however, in contrast, rupture caused by deceleration or hydrostatic forces may occur with minimal or no apparent thoracic trauma.^19-21^ Besides, atrial injury is more commonly associated with deceleration or hydraulic forces, whereas ventricular rupture is primarily caused by compression.^19^

 The overall mechanisms of acute BCR are as follows: (1) rapid deceleration causing the heart to rotate anteriorly, pulling away from the relatively fixed great vessels (venae cavae, aorta, pulmonary arteries, and veins)^12^; (2) The transfer of substantial blunt force from the abdomen or lower limbs through the venous system to the heart chambers, commonly referred to as the “hydraulic effect.”,^8,22^ and (3) Rupture of the heart as a result of being compressed between the sternum and the vertebral column.^12,23^ As mentioned earlier, both autopsy and clinical studies show a higher incidence of right-sided injuries, potentially due to the relative thickness of the walls.^8,20,24-26^ Additionally, since most BCRs result from anterior rather than posterior chest trauma, and given that anterior chest trauma is more common in traffic accidents, the anatomical likelihood of rupture is greater in the right atrium and right ventricle compared to the left ventricle and left atrium.^27-31^

 We did not observe any traumatic lesions during the external examination of the chest region in the current case report. No fractures were detected in the chest cage bones (sternum, ribs); neither was there any bruising or bleeding in the soft tissues during the internal examination. Additionally, no bruising was noted in the pericardium, which was found to be intact. However, upon incision of the pericardium, approximately 400 cc of coagulated blood and a 2-cm rupture in the right ventricle were observed. While cardiac tamponade is most often the result of a penetrating chest injury, it can also occur due to blunt chest trauma. The fibrous construction of the pericardium and weakness of the smooth type of myocardial muscle fibres may cause the cardiac walls to rupture along with leakage of blood into the pericardium.^32,33^

 We hypothesize that in the current case, when the individual suddenly hit the steering wheel with his chest, the heart, which is connected to the large vessels in the chest cavity and was filled with blood, was suddenly squeezed between the spine at the back and the sternum in the front. This led to the rupture of the right ventricle and can be likened to the rupture of a water-filled balloon when it is suddenly squeezed between two hands.

 RuDusky and Basil proposed the following classification for blunt cardiac trauma: *stages 0 (suspect), I (mild), II (moderate), III (severe), and IV (catastrophic)*. The current case showed ‘myocardial rupture’, which is compatible with grade IV blunt cardiac trauma (catastrophic-* Permanent Sequelae or Death*).^34^ The American Association for the Surgery of Trauma (AAST) has also published a grading scale for cardiac injuries, categorizing them as pericardial, myocardial, coronary artery, or electrical conduction system injuries^31,35,36^ (Table 1). Viano and colleagues have also described the probability of chest injury with an abbreviated injury score (AIS) of 4 or greater, based on the viscous response. These authors have noted that the chest appears to be more susceptible to viscous trauma than the abdomen.^37^ Consistent with previous reports, the anatomical region of cardiac injury and the presence of cardiac tamponade in the current case was classified as Grade IV on the aforementioned grading scale.

**Table 1:** American Association for the Surgery of Trauma Cardiac Injury Scale

**Grade I**
1. Blunt cardiac injury with minor Electrocardiogram abnormality (non-specific ST or T wave changes, premature atrial or ventricular contractions, or persistent sinus tachycardia
2. Blunt or penetrating pericardial wound without cardiac injury, tamponade, or cardiac herniation
**Grade II**
1. Blunt cardiac injury with heart block or ischemic changes without cardiac failure
2. Penetrating tangential cardiac wound, up to but not extending through endocardium, without tamponade
**Grade III**
1. Blunt cardiac injury with sustained or multifocal ventricular contractions
2. Blunt or penetrating cardiac injury with septal rupture, pulmonary or tricuspid incompetence, papillary muscle dysfunction, or distal coronary artery occlusion without cardiac failure
3. Blunt pericardial laceration with cardiac herniation
4. Blunt cardiac injury with cardiac failure
5. Penetrating tangential myocardial wound, up to but not through endocardium, with tamponade
**Grade IV**
1. Blunt or penetrating cardiac injury with septal rupture, pulmonary or tricuspid incompetence, papillary muscle dysfunction, or distal coronary artery occlusion producing cardiac failure
2. Blunt or penetrating cardiac injury with aortic or mitral incompetence
3. Blunt or penetrating cardiac injury of the right ventricle, right or left atrium
**Grade V**
1. Blunt or penetrating cardiac injury with proximal coronary artery occlusion
2. Blunt or penetrating left ventricular perforation
3. Stellate injuries, less than 50% tissue loss of the right ventricle, right or left atrium
**Grade VI**
1. Blunt avulsion of the heart
2. Penetrating wound producing more than 50% tissue loss of a chamber

 Toxicological analysis of the current case revealed 950 ng/mL of pregabalin in blood samples taken during the preliminary examination at the hospital morgue, and 660 ng/mL in blood samples collected during the autopsy. These levels are below the lethal dose. Nonetheless, considering the findings from the crime scene investigation such as the absence of any obvious road hazards, lack of rainy weather, or any other vehicles in the crash, we suspect that the sedative effects of pregabalin misuse might have contributed to the crash. During the crime scene investigation, it was noted that the individual was not wearing the seatbelt; additionally, the bottom part of the steering wheel was broken and bent inward. We believe that the individual may have struck his chest on the steering wheel, leading to blunt trauma; lack of seatbelt protection is also likely to have contributed to this trauma.

## Conclusion

 The current case underscores the critical importance of recognizing and addressing BCIs in trauma cases, particularly in the context of one-sided motor vehicle collisions. Although BCR remains a rare occurrence, its high mortality rate necessitates heightened awareness and preparedness among emergency responders. We suggest that forensic pathologists should consider the occurrence of BCR even in the absence of chest trauma or visible injuries to the chest wall, especially in overweight individuals, emphasizing the need for thorough internal examinations to identify such injuries.
